# Awards and Criticisms

**Published:** 1888-07-28

**Authors:** 


### THE METROPOLITAN HOSPITAL SUNDAY FUND, 1888.

### AWARDS AND CRITICISMS.

THE Committee of Distribution have adopted the following report to the Council, which will meet on Tuesday, the 31st inst., to receive it:—

The total amount available for distribution, after allowing sufficient for liabilities and the usual current expenses, is £39,321 17*s*. 6*d*. Of this total £37,721 17*s*. 6*d*. is now recommended to 107 hospitals and 50 dispensaries.

Four per cent. of the total collected—viz., £1,600—is set apart to purchase surgical appliances, in obedience to Law XII., in monthly proportions during the ensuing year.

Your committee recommend that all payments to the fund after this date be carried to the credit of next year's fund.

In compliance with an order of the Council, and for the special use of its members, tables of statistics have been prepared as usual, showing an analysis of the number of beds in hospitals, the cost of patients both at hospitals and dispensaries, the proportionate expense of management, as well as other valuable information, and copies are now sent to every member of the Council.

The number of deputations representing committees of various hospitals, invited to confer with your committee, and to offer explanations on matters of apparently unsatisfactory character, was eleven. Five of these deputations attended, and in two cases your committee regret to report that they are not prepared to recommend any award after these interviews. Five institutions sent replies but did not attend, and one application was withdrawn.

One of our old established lying-in hospitals, not being in immediate want of funds, made no application this year.

Four institutions which have been less than three years established, and hence could not comply with Law IV. by furnishing balance-sheets for three years, had to be refused. One application, from an institution being neither hospital nor dispensary, was refused; and one came too late.

Your Committee regret to add that they have felt it their duty to make no award to one dispensary which applied this year for the first time, owing to unsatisfactory and high rate of management.

Guy's Hospital has, for the first time since the establishment of this fund, made application to participate. Your Committee have examined very carefully into the accounts and the present needs of this institution during several of their sittings, and have recommended an award which they hope will be approved by the Council.

The award, if any, to St. John's Hospital for Diseases of the Skin is held in abeyance, litigation having arisen in connexion with the management.

The following one hundred and one hospitals, seven institutions, and fifty dispensaries, making a total of 158, the same number as last year, but an increase of fifty-three institutions since 1873, will participate to the extent stated.

### PARTICULARS OF THE AWARDS FOR 1888.

### TWENTY-TWO GENERAL HOSPITALS, VIZ:

Charing Cross, West Strand, W.C., £989 11*s*. 8*d*.; French, Lisle Street, W.C., £208 6*s*. 8*d*.; German, Dalston Lane, E., £677 1*s*. 8*d*.; Great Northern Central, Caledonian Road, N., £270 16*s*. 8*d*.; Guy's, £520 16*s*. 8*d*.; Italian, Queen Square, W.C., £72 18*s*. 4*d*.; King's College, Lincoln's Inn Fields, W.C., £[illegible],354 3*s*. 4*d*.; London, Whitechapel, E., £3,333 6*s*. 8*d*.; Metropolitan, Kingsland Road, N., £208 6*s*. 8*d*.; Miller Hospital and Royal Kent Dispensary, £[illegible]86 9*s*. 2*d*.; North-West London, Kentish Town Road, N.W., £291 1[illegible]*s* 4*d*.; Poplar, East India Road, E., £218 15*s*.; Royal Free, Gray's Inn Road, W.C., £958 6*s*. 8*d*.; St. George's, Hyde Park Corner, S.W., £1,562 10*s*.; SS. John and Elizabeth, Great Ormond Street, W.C., £[illegible]04 3*s*. 4*d*.; St. Mary's, Paddington, W., £1,770 16*s*. 8*d*.; Seamen's, Greenwich, S.E., £625; The Middlesex, Charles Street, W., £2,031 5*s*.; Tottenham Training Hospital, Tottenham, N., £260 8*s*. 4*d*.; University College, Gower Street, W.C., £1,354 3*s*. 4*d*.; West London, Hammersmith, W., £520 16*s*. 8*d*.; Westminster, Broad Sanctuary, S.W.; £1,041 13*s* 4*d*.

### SPECIAL HOSPITALS.

FIVE CHEST HOSPITALS.—City of London Hospital for Diseases of the Chest, Victoria Park, E., £885 8*s*. 4*d*.; Hospital for Consumption, Brompton, S.W., £1,875; North London Consumption Hospital, Hampstead, £208 6*s*. 8*d*.; Royal Hospital for Diseases of the Chest City Road, £312 10*s*; Royal National Hospital for Consumption (Ventnor), £[illegible]08 6*s* 8*d*.

TWELVE CHILDREN'S HOSPITALS.—Alexandra, Queen Square, W.C., £177 1*s* 8*d*.; Belgrave, 1, Cumberland Street, Pimlico, S.W., £135 8*s*. 4*d*.; Cheyne Hospital for Children, Chelsea, S.W., £109 7*s*. 6*d*.; East London, Shadwell, E., £416 13*s*. 4*d*.; Evelina, Southwark Bridge Road, S.E., £458 6*s*. 8*d*.; Home for Children, Maida Vale, N.W., £5[illegible] 1*s*. 8*d*.; Home for Sick Children and South London Dispensary for Women, Sydenham, S.E., £145 16*s*. 8*d*.; Hospital for Sick Children, Great Ormond Street, W.C., £729 3*s* 4*d*.; North Eastern, 327, Hackney Road, E., £333 6*s*. 8*d*.; Paddington Green Hospital for Sick Children, £[illegible]66 13*s*. 4*d*.; Victoria, Queen's Road, Chelsea, S.W., £333 6*s*. 8*d*; "The Vine," Sevenoaks, £26 0*s*. 10*d*.

THREE LYING-IN HOSPSTALS.—British, Endell Street, W.C., £62 [illegible]0*s*.; General, York Road, Lambeth, S.E., £93 15*s*.; Queen Charlotte's, Marylebone Road, W., £312 10*s*.

SIX HOSPITALS FOR WOMEN.—Chelsea Hospital for Women, Fulham Road, S.W., £192 14*s*. 2*d*; Hospital for Women, Soho Square, W., £427 1*s* 8*d*.; Grosvenor Hospital for Women and Children Vincent Square, S.W., £83 6*s*. 8*d*.; New Hospital for Women, 322, Marylebone Road, W., £125; Royal Hospital for Diseases of Children and Women, Waterloo Bridge Road, S.E., £218 15*s*.; Samaritan Free Lower Seymour Street, W., £479 3*s*. 4*d*.

### TWENTY-FOUR OTHER SPECIAL HOSPITALS.

Cancer, Brompton, S.W., £468 15*s*.; London Fever, Liverpool Road, N., £5[illegible]2 18*s*. 4*d*.; St. Mark's (Fistula), City Road, E.C., £93 15*s*.; National, for Diseases of the Heart, Soho Square, W., £140 12*s*. 6*d*.; Female Lock, Westbourne Green, W., £26[illegible] 8*s* 4*d*.; Male Lock, Dean Street, Soho, W., £72 18*s*. 4*d*.; Hospital for Epilepsy, Paralysis and other Diseases of the Nervous System, Portland Terrace, N.W., £83 6*s*. 8*d*; National, for the Paralyzed and Epileptic, Queen Square, W.C., £416 13*s*. 4*d*.; Central London Ophthalmic, Gray's Inn Road, W.C., £41 13*s*. 4*d*; Royal London Ophthalmic, Moorfields, E.C., £572 18*s* 4*d*.; Royal South London Ophthalmic, St. George's Circus, S.E., £93 15*s*.; Royal Westminster Ophthalmic, King William Street, W.C., £88 10*s*. 10*d*.; Western Ophthalmic, Marylebone Road W., £30 4*s*. 2*d*.; City Orthopædic, Hatton Garden, E.C., £78 2*s* 6*d*.; National Orthopædic, Great Portland Street, W., £31 5*s*.; Royal Orthopædic, Hanover Square W., £88 10*s*. 10*d*.; Hospital for Diseased Skin, Stamford Street, S.E., £31 5*s*; St. John's, for Diseased Skin, Leicester Square, W.C., deferred pending litigation; Central London Throat and Ear, Gray's Inn Road, W.C., £57 5*s*. 10*d*.; Hospital for Diseases of the Throat, Golden Square, W. £36 9*s*. 2*d*.; London Homœopathic, Great Ormond Street, W.C., £208 6*s*. 8*d*.; London Temperance, Hampstead Road, N.W., £364 11*s*. 8*d*; The Dental, Leicester Square, W.C., £83 6*s*. 8*d*.; National Dental, 149, Great Portland Street, W., £17 14*s*. 2*d*.

### EIGHTEEN CONVALESCENT HOSPITALS.

Metropolitan Convalescent Institution, Walton-on-Thames, &c., £677 1*s* 8*d*; Bexhill-on-Sea Convalescent Home, £208 6*s*. 8*d*.; All Saints' Convalescent Home, Eastbourne, £598 19*s* 2*d*.; Mrs Gladstone's Convalescent Home Woodford, E., £208 6*s* 8*d*.; Hanwell Convalescent Home, Middlesex, £26 0*s*. 10*d*; Herbert Convalescent Hospital, Bournemouth, £20 16*s*. 8*d*; King's College Convalescent Hospital, Hemel Hempstead, £125; Mrs. Kitto's Convalescent Hospital, Reigate, Surrey, £72 18*s*. 4*d*.; Mrs. Masham's Convalescent Hospital, Brighton £83 6*s*. 8*d*; Mary Wardell Convalescent Home, Great Stanmore, £125; Morley Convalescent Hospital, St. Margaret's, Kent, £5[illegible] 1*s*. 8*d*.; St. Andrew's Convalescent Hospital, Clewer, £145 16*s*. 8*d*.; St. Andrew's Convalescent Hospital, Folkestone, £14[illegible] 16*s*. 8*d*.; St. Leonards-on-Sea Convalescent Hospital for Children, £104 3*s*. 4*d*.; St. Mary Magdalene Convalescent Home, Paddington, W., £52 1*s*. 8*d*.; Seaside Convalescent Hospital, Seaford, £104 3*s*. 4*d*.; Children's Convalescent Homes, Bexhill and Ramsgate, £53 2*s*. 6*d*.; Princess Frederica's Convalescent Home, East Moulsey, £41 13*s*. 4*d*.

### ELEVEN COTTAGE HOSPITALS.

Beckenham, £46 17*s* 6*d*.; Blackheath and Charlton, £67 14*s*. 2*d*.; Burstead, £[illegible]0 16*s*. 8*d*.; Chislehurst, etc., £52 1*s*. 8*d*.; Eltham £36 9*s*. 2*d*.; Enfield, £36 9*s*. 2*d*.; Epsom and Ewell, £41 13*s*. 4*d*.; Reigate and Redhill, £72 18*s*. 4*d*.; Shedfield, £10 8*s*. 4*d*.; Sidcup, £26 0*s*. 10*d*; Wimbledon, £3[illegible] 9*s*. 2*d*.

### SEVEN INSTITUTIONS.

Establishment for Gentlewomen, Harley Street, W., £83 6*s*. 8*d*.; Hampstead Home Hospital, £52 1*s*. 8*d*.; National Sanatorium Bournemouth, £72 18*s*. 4*d*.; Royal Sea Bathing Infirmary, Margate, £572 18*s*. 4*d*; Invalid Asylum, Stoke Newington, £72 18*s*. 4*d*.; "Firs Home," Bournemouth, £20 16*s*. 8*d*.; "St. Catherine's Home," Ventnor, £46 17*s*. 6d.

### FIFTY DISPENSARIES.

Battersea (Provident), £57 5*s*. 10*d*.; Brixton, &c., £46 17*s*. 6*d*.; Brompton (Provident), £22 18*s* 4*d*.; Camberwell Provident), £93 15*s*.; Chelsea, £46 17*s*. 6*d*; Child's Hill (Provident), £13 10*s*. 10*d*.; City, £119 15*s*. 10*d*.; City of London and East London, £18 15*s*; Clapham (General), £36 9*s* 2*d*.; Eastern, £31 5*s*.; Farringdon, £52 1*s* 8*d*; Finsbury £55 4*s* 2*d*.; Forest Hill, £36 9*s*. 2*d*.; Gipsy Hill (Provident), £[illegible]0 16*s* 8*d*.; Hampstead (Provident) £46 17*s*. 6*d*.; Holloway and North Islington, £88 10*s*. 10*d*.; Islington, £62 10*s*.; Kensington, £83 6*s*. 8*d*.; Kilburn, &c., £50; Kilburn (Provident), £31 5*s*.; London, Spitalfields, E., £20 16*s* 8*d*.; Margaret Street Infirmary for Consumption, W., £17 14*s* 2*d*.; Metropolitan (Fore Street, E.C.) £[illegible]1 13*s*. 4*d*; Notting Hill (Provident), £26 0*s*. 10*d*.; Paddington (Provident) £41 13*s* 4*d*.; Portland Town (Free), £20 16*s*. 8*d*.; Portobello Road (Provident) £5 4*s* 2*d*.; Public, Stanhope Street, W.C.; £52 1*s*. 8*d*; Queen Adelaide's, Bethnal Green, E., £46 17*s*. 6*d*.; Royal General, Bartholomew Close, E.C., £62 10*s*.; Royal Maternity Charity, Finsbury Square, E.C., £114 11*s*. 8*d*.; Royal Pimlico (Provident), £52 1*s*. 8*d*.; Royal South London, St. George's Circus, S.E., £62 10*s*; SS. George's and James's, King Street, W., £67 14*s* 2*d*.; St. George's, Hanover Square (Provident), £52 1*s*. 8*d*.; St. John's Wood (Provident), £26 0*s* 10*d*.; St. Marylebone, General £41 13*s*. 4*d*; St. Pancras and Northern, £37 10*s*.; SS. Paul and Barnabas (Provident) Pimlico, S.W., £46 17*s*. 6*d*.; South Lambeth, £46 17*s* 6*d*.; South London Medical Aid Institute, Waterloo Bridge Road, S.E., £36 9*s*. 2*d*.; Stamford Hill, etc., Stoke Newington, N., £54 3*s*. 4*d*.; Tower Hamlets, Stepney, E., £41 13*s*. 4*d*.; Wandsworth Common (Provident), £10 8*s* 4*d*; Westbourne (Provident), £20 16*s*. 8*d*.; West Dulwich (Provident), £7 5*s*. 10*d*; Western, Rochester Row, Westminster, S.W., £52 1*s*. 8*d*.; Western General, Marylebone Road, S.W., £128 2*s*. 6*d*.; West Ham and Stratford, E., £62 10*s*; Westminster General, Gerrard Street, W., £33 6*s*. 8*d*.

Three hundred and forty pounds in all will be deducted from certain of the above sums, being the amounts received from collections sent direct to particular institutions.

